# Variability in baseline travel behaviour as a predictor of changes in commuting by active travel, car and public transport: a natural experimental study

**DOI:** 10.1016/j.jth.2015.11.002

**Published:** 2016-03

**Authors:** Eva Heinen, David Ogilvie

**Affiliations:** a MRC Epidemiology Unit and UKCRC Centre for Diet and Activity Research (CEDAR), School of Clinical Medicine, University of Cambridge, Box 285, Cambridge Biomedical Campus, Cambridge CB2 0QQ, United Kingdom; b Delft University of Technology, Faculty of Technology, Policy & Management, Department of Transport & Logistics, Jaffalaan 5, 2628 BX Delft, The Netherlands

**Keywords:** Behaviour change, Mode choice variability, Active travel, Public transport, Car use, Longitudinal

## Abstract

**Purpose:**

To strengthen our understanding of the impact of baseline variability in mode choice on the likelihood of travel behaviour change.

**Methods:**

Quasi-experimental analyses in a cohort study of 450 commuters exposed to a new guided busway with a path for walking and cycling in Cambridge, UK. Exposure to the intervention was defined using the shortest network distance from each participant’s home to the busway. Variability in commuter travel behaviour at baseline was defined using the Herfindahl–Hirschman Index, the number of different modes of transport used over a week, and the proportion of trips made by the main (combination of) mode(s). The outcomes were changes in the share of commute trips (i) involving any active travel, (ii) involving any public transport, and (iii) made entirely by car. Variability and change data were derived from a self-reported seven-day record collected before (2009) and after (2012) the intervention. Separate multinomial regression models were estimated to assess the influence of baseline variability on behaviour change, both independently and as an interaction effect with exposure to the intervention.

**Results:**

All three measures of variability predicted changes in mode share in most models. The effect size for the intervention was slightly strengthened after including variability. Commuters with higher baseline variability were more likely to increase their active mode share (e.g. for HHI: relative risk ratio [RRR] for interaction 3.34, 95% CI 1.41, 7.89) and decrease their car mode share in response to the intervention (e.g. for HHI: RRR 7.50, 95% CI 2.52, 22.34).

**Conclusions:**

People reporting a higher level of variability in mode choice were more likely to change their travel behaviour following an intervention. Future research should consider such variability as a potential predictor and effect modifier of travel and physical activity behaviour change, and its significance for the design and targeting of interventions.

## Highlights

• Changing the environment can promote physical activity by altering travel behavior.
• Travel mode choice can be highly variable over the course of a week in some commuters.
• Changes in travel behaviour were associated with baseline variability in mode choice.
• Active travel increased with higher levels of intervention exposure and variability.
• Including variability in models slightly strengthened intervention effect estimates.

## 1 Introduction

Changes in mode of transport have the potential to increase levels of population health. A reduction in car travel may reduce carbon emissions and injuries (Woodcock et al., 2009), whereas active travel – walking and cycling – is associated with higher levels of physical activity and can provide a sufficient level of activity to contribute to health gain (Chief Medical Office, 2011).

Studies in various domains of health-related behaviour change suggest that existing behaviour predicts future behaviour. For example, the number of cigarettes smoked by an individual predicts the likelihood of smoking cessation (Hymowitz et al., 1997) and alcohol intake in adolescence is correlated with alcohol intake in adulthood (Paavola et al., 2004). Recent studies of active travel have found that time spent in active commuting at baseline is associated with changes in active commuting time (Panter et al., 2016), that the mode of transport used for commuting at baseline is associated with changes in the shares of trips made by active travel and by car (Heinen et al., 2015a), and that mode choice at baseline predicts the use of new transport infrastructure (Goodman et al., 2013). More generally, habits of using a specific mode of transport are a strong correlate of (active) travel behaviour and thought to hinder behaviour change (De Bruijn and Gardner, 2011, Aarts et al., 1998, Bamberg and Schmidt, 2003, Gärling and Axhausen, 2003).

However, characteristics of travel behaviour other than the baseline value of the outcome of interest may be at least as strong a predictor of behaviour change. In particular, baseline variability of mode choice, which is in a way the opposite of habit, may correspond with a higher inclination to change. Here, we use ‘variability’ to refer to the level of variation in modes of transport used by an individual within a certain period, and ‘change’ to refer to a shift towards or away from a given mode of transport over time.

Several theories and models have expressed the idea that behaviour change involves moving through different phases. A commonly applied model is the transtheoretical model (Prochaska et al., 1992, Prochaska and Velicer, 1997), although other stage-based models may be more appropriate for a given type of behaviour (Sutton, 2002). For example, Jones and Sloman (2003) describe a model with seven phases: awareness of a key issue, acknowledging relevance, perception of options, evaluation of options, making a choice, experimental behaviour, and habitual behaviour. This conceptualisation assumes that a period of experimental behaviour precedes the establishment of new or even habitual behaviour. In this context baseline variability may be seen as a characteristic of an experimental phase which precedes a phase of more established behaviour.

Baseline variability may also increase an individual’s self-efficacy to use particular modes of transport. Self-efficacy refers to confidence in the ability to perform a certain behaviour and is thought to drive behaviour change (Bandura, 1986, Strecher et al., 1986). Variability in mode choice at baseline may therefore correspond with higher levels of self-efficacy to use several different modes of transport.

Not all environments are equally supportive for all modes of transport. Cross-sectional studies have shown that characteristics of the built environment are associated with differences in travel behaviour (Handy et al., 2002, Ewing and Cervero, 2010), and a small number of more recent intervention studies (e.g. Goodman et al., 2014; Hooper et al., 2014) provide stronger evidence for causal effects of environmental changes on travel behaviour (National Institute for Health and Care Excellence (Nice), 2014, McCormack and Shiell, 2011). It is conceivable that individuals with greater baseline variability in commute mode choice may be more likely to change their behaviour in response to environmental changes than those who show less variability at baseline.

We aimed to increase our understanding of the relationship between baseline behaviour and behaviour change over time, using the opportunity presented by an intervention study to discriminate between variability and change. Previous results from our own natural experimental study have shown that changing the built environment by constructing new transport infrastructure can result in changes in mode choice (Heinen et al., 2015a) and use (Panter et al., 2016) in commuters. In this paper, we investigate whether variability in mode choice at baseline increased the likelihood of change in the share of commuting trips made by different modes of transport, both independently and as a modifier of the effect of exposure to the intervention. An independent effect would indicate that indivdiuals who are more variable have a higher likelihood of changing their travel behaviour. A significant interaction effect would indicate that if individuals are more exposed to an intervention, they have a higher likelihood of changing if they are also more variable in their baseline behaviour – in other words, that individuals who are more variable may be more sensitive to interventions. We tested the effect of variability in an intervention study to discriminate between variability (short or long-term random changes) and change (non-random change in behaviour).

## 2 Methods

### 2.1 Setting

Data were collected in Cambridgeshire, UK (123,900 inhabitants) (ONS, 2011). The city of Cambridge has a comparatively affluent and well-educated population, and 28% of commuting trips are made by bicycle (ONS, 2011).

### 2.2 Intervention

**Fig. 1:**
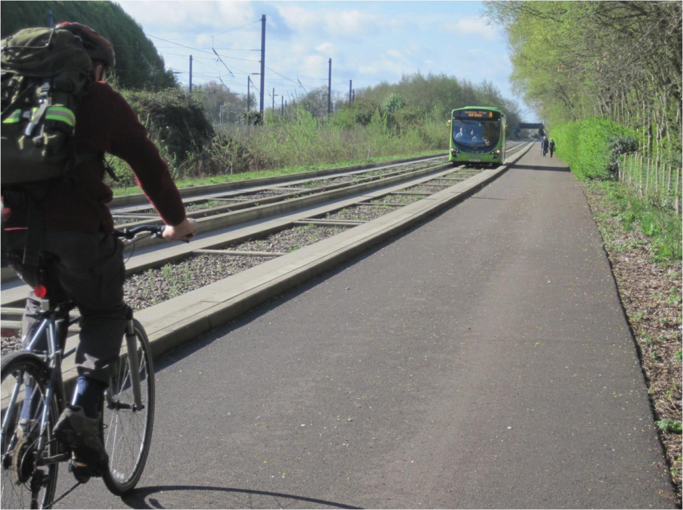
The Cambridgeshire guided busway.

The Cambridgeshire Guided Busway (hereafter referred to as ‘the busway’) was officially opened in August 2011 (Fig. 1). It consists of a 25 km guideway (separate off-road track) for buses; a service path, which can be used for walking and cycling; and three park-and-ride sites. The busway passes close to several major employment sites in the city centre and urban fringes, and was implemented to reduce traffic congestion on the roads around Cambridge (Atkins, 2004).

### 2.3 Data collection and study sample

Questionnaire data were collected by post annually between 2009 and 2012 as part of a natural experimental cohort study. At the time of recruitment, participants were 16 years of age or over, working in areas of Cambridge to be served by the busway and living within approximately 30 km of the city centre (Ogilvie et al., 2010). To avoid biasing recruitment and responses, the study was presented to participants as a study of ‘commuting and health’ and the aim of evaluating the busway was not made explicit.

The analyses reported in this paper used the first (pre-intervention in 2009) and fourth (final, post-intervention in 2012) survey waves. The busway was partly implemented during the second and third waves of data collection, and these intermediate waves were therefore not analysed in this paper. 1164 participants took part in the first wave, of whom 500 (43%) also took part in the fourth wave. The Hertfordshire Research Ethics Committee approved the study and the baseline data collection (reference number: 08/H0311/208) and the Cambridge Psychology Research Ethics Committee granted approval for the follow-up data collection used in this analysis (reference number: 2014.14). All participants provided written informed consent.

### 2.4 Exposure to the intervention

We derived an objective measure of exposure to the intervention for each individual, based on the proximity of the postcode of their home address at baseline to the nearest busway stop or path access point – whichever was nearer – because respondents could have been exposed by using the guided bus or by using the path for walking or cycling (Heinen et al., 2015b). We applied a negative square root transformation to the distance, so that greater proximity corresponded with a higher level of exposure to the intervention. This produced comparable, but slightly more conservative and more easily interpretable, model outputs to those produced by a log transformation, the most obvious alternative.

### 2.5 Outcomes

Changes in mode shares for commuting trips were calculated between the pre- and post-intervention surveys using a self-reported seven-day travel record (Panter et al., 2011). For each day, respondents were asked to report the day of the week and their mode(s) of travel to and from work, or to positively indicate that they had not travelled to work that day. We truncated the travel diary to the first seven reported consecutive days if more days were reported. We excluded respondents who had returned a blank travel diary in either wave (*n*=28) or had accounted for fewer than three days of the week (*n*=3). No imputations were made if travel data appeared incomplete. Individuals reporting fewer or greater than seven consecutive days, failing to report an apparently missing trip to or from work (unless at the beginning or end of the reporting period), or failing to report the day of the week were included in the main analysis but excluded from a sensitivity analysis (sensitivity test s4a, see below).

We derived three specific mode share outcomes. These were changes in the proportion of commute trips (i) involving any active travel, (ii) involving any public transport, and (iii) made entirely by car, reflecting the aims and nature of the intervention (Table 1) and corresponding with other analyses (Heinen et al., 2015). The changes in mode share were classified into three groups: decrease, no change and increase. For the main analysis, only changes of greater than 20% were classified as an ‘increase’ or a ‘decrease’. Differences of this magnitude represent a change of behaviour affecting more than one day a week (assuming a five-day working week) and are therefore more likely to capture true change instead of variability (Table 1). In addition, we conducted sensitivity tests using a less and a more restrictive definition of change, the first using a threshold of 0% and the second using a threshold of 30%.

### 2.6 Variability

Using the seven-day travel record in the pre-intervention survey, we derived five measures of modal variability. These included two measures of variability at stage level (a stage is a part of a trip; for example a trip involving both cycling and bus travel would comprise two stages, one by bicycle and one by bus) and three measures of variability at trip level (based on the mode or combination of modes of transport used for the entire trip). Variability at stage level reflects the variation in all possible modes used, independent of whether these are used in combination with other modes and independent of the length of each stage. Variability at trip level reflects the variation in the mode or combination of modes used between trips, and considers each combination of modes as a unique choice.

The two measures of variability at stage level considered were (1) the Herfindahl–Hirschman Index (HHI) and (2) entropy. The HHI is a measure of market concentration and is one of the most commonly applied measures of mode choice variability (Susilo and Axhausen, 2014, Heinen and Chatterjee, 2015). It is calculated as the sum of the squared values (S) of the share of each mode within all commuting trips (Rhoades, 1993) (Eq. (1)). A normalised index ranges from 0 to 1, where the closer to 1 the more one mode dominates the travel of an individual (Eq. (2)). Entropy is calculated as the sum of the negative value of the chance that a mode is used (the number of stages on which a specific mode is used divided by the total number of stages), multiplied by the log_2_ of this chance (Eq. (3)). We considered the following six modes for the calculation of the HHI and entropy: car, bicycle, walking, bus, train and other. For variability at trip level we calculated (3) a count variable representing the number of modes or combinations of modes used for trips to work, (4) a binary variable indicating whether more than one mode or combination of modes was used for trips to work, and (5) the proportion (share) of trips to work made by the most commonly reported mode or combination of modes for a given individual. To calculate these variables we considered the same six modes, as well as any combinations of these (such as car plus walking, or bus plus cycling).

(1) $$H\;H\;I_{basic}=\sum_{i=1}^{N}s_{i}^{2}$$

(2) $$H\;H\;I=\frac{H\;H\;I_{basic}-1/N}{\left(1-1/N\right)}$$

(3) $$Entrophy=\sum_{i=1}^{N}-s*\log_{2}(S)$$

The HHI and entropy indicators were highly correlated (0.99), and the HHI was selected for analysis as the calculation and interpretation were easier. The three trip-level indicators were also closely related; of these, we used the pair of quasi-continuous (count and proportion) variables that also showed less correspondence with each other than the third (binary) measure. The HHI and the mode share (proportion) of the most commonly used mode or combination of modes were transformed in order that an increase on all indicators corresponded with higher levels of variability (Tables 2 and 3).

### 2.7 Covariates

The following covariates, ascertained at baseline using questionnaire items reported previously (Panter et al., 2011), were included in our analysis: gender, age, education level, car ownership, housing tenure, possession of a driving licence, access to a bicycle, presence of children in the household, difficulty walking, commute distance, two variables indicating that individuals had moved home or work location, availability of (free) parking at work, and Mental Component Summary (MCS-8) and Physical Component Summary (PCS-8) scores representing respondents’ health-related quality of life derived from the Medical Outcomes Study Short Form (SF-8) (Ware et al., 2008); along with an indicator of residential settlement size – the urban/rural classification of the census output area of each participant’s home postcode (Bibby and Shephard, 2004) (Table 4).

### 2.8 Analysis

Several multinomial logistic regression models were estimated to test the association of variability with changes in commute mode shares, to test whether variability predicted behaviour change either independently or as an interaction effect. We progressively adjusted the models by initially estimating the effect of (1a) only baseline variability on changes in mode share, followed by (2a) adding exposure to the intervention to the model. Then (3a) an interaction term between exposure to the intervention and baseline variability of mode choice was added, which was calculated by multiplying the adjusted means. Finally the (4a) maximally adjusted model was estimated, including baseline variability, exposure to the intervention, the interaction term between variability and exposure to the intervention (step 3a) if significant (*p*<0.05), and the covariates. Age and gender were always included, and the other covariates were included only if associated with the outcome at *p*<0.25 in unadjusted models.

We tested the stability of the maximally adjusted models (4a) by performing sensitivity tests. These involved applying either (s1a) the less restrictive (0%) or (s2a) the more restrictive (30%) definition of change, and repeating the maximally adjusted models restricted to either (s3a) individuals who had not moved home or work location or (s4a) those who had ‘perfectly’ reported their weekly travel record.

In addition, we tested whether including a measure of variability changed the estimated effect of exposure to the intervention. To test whether variability was a confounder, we progressively adjusted our models by first (1b) including only exposure to the intervention, then (2b) adding baseline variability. We then proceeded to maximal adjustment by including all covariates as in step 4a, but (3b) without including the interaction between exposure to the intervention and variability and then (4b) without either the interaction or the measure of variability. This progressive adjustment enabled us to investigate the extent to which variability confounded the estimated effect size for exposure to the intervention.

## 3 Results

Below we report the results from the maximally adjusted models. The results of interim adjustments and sensitivity tests are presented in the appendices.

### 3.1 Change in share of commute trips involving any active travel

Variability in baseline mode choice predicted a change in the share of trips involving any active travel in both directions. Higher levels of variability in all indicators predicted a decrease in active travel share (for HHI: relative risk ratio [RRR]: 40.82, 95% confidence interval (95% CI): 10.63–156.70; for number of modes: 3.23, 2.04–5.12; for proportion main mode: 46.32, 8.10–264.90), as well as an increase in active travel share (for HHI: 5.48, 1.71–17.52; for number of modes: 2.95, 1.81–4.79; for proportion main mode: 106.40, 14.97–756.40) (Table 5). By way of illustration, this indicates that individuals who had a 0.1 higher value in their HHI (which could be a result of many combinations, for example the difference between using the car for 70% of trips and the bike for 30% on the one hand, and a 50/50 split on the other) were four times as likely to reduce their active travel share over time. Similarly, those who had one additional mode of transport in their baseline modal mix were more than three times as likely to reduce their active travel, and those who relied on their main mode for 90% rather than 100% of their trips were 4.6 times as likely to reduce their active travel. Taken together, these results indicate that individuals with a higher level of variability at baseline were more likely to change the share of their trips involving any active travel over time.

Exposure to the intervention was associated with an increase, but not with a decrease, in active travel share in the fully adjusted models. The results correspond with individuals living, for example, 4 km from the busway being from 60% to 70% more likely (depending on the indicator of variability) to have increased their active travel share than those living 9 km away^1^.

The interaction terms between variability and exposure to the intervention predicted a significant increase in active travel share for two indicators (HHI and proportion main mode). Individuals who were more exposed to the busway were thus more likely to increase their active travel share when they had higher levels of variability at baseline. The interaction terms were not associated with a decrease in active travel mode share.

As expected, the effect of variability was larger in the models that used a less restrictive definition of change over time. The sensitivity test using a more restrictive definition of change and stage-level variability (HHI) no longer predicted a decrease in active travel, but the associations for all other predictors and the directions of the estimated effect sizes remained similar (Appendix A1). The effect of exposure to the intervention was slightly strengthened in all models that included baseline variability (Appendix A2).

### 3.2 Change in share of commute trips involving any public transport

Baseline variability of mode choice was associated with changes in the share of commute trips involving any public transport in both directions in most models. The HHI was non-significantly associated with an increase in public transport, but the other two indicators were significantly associated with an increase in public transport share (number of modes: 3.64, 2.17–6.11; proportion main mode: 50.50, 7.14–357.30) and all indicators of variability predicted a decrease in public transport share (HHI: 82.83, 15.17–452.40; number of modes: 1.78, 1.09–2.10; proportion main mode: 8.29, 1.18–58.47) (Table 6). This indicates that variability is also a predictor of some changes in the share of trips involving any public transport.

Exposure to the intervention was associated with an increase, but not a decrease, in public transport mode share. The results correspond with individuals living, for example, 4 km from the busway being 50% more likely to have increased their share of trips involving some public transport than those living 9 km away.

The interaction term between variability and exposure to the intervention was not significant in the unadjusted models.

In some sensitivity tests, both variability and exposure to the intervention were no longer significantly associated with the outcome, although the direction of the effect remained similar (Appendix B1). The effect of exposure to the intervention was hardly affected by including variability (Appendix B2); in general this association was strengthened for an increase, and attenuated for a decrease, in public transport mode share.

### 3.3 Change in share of commute trips made only by car

Variability in mode choice at baseline predicted changes in car share. All indicators of variability were significantly associated with a decrease in car share (HHI: 6.00, 1.51–23.79; number of modes: 3.31, 1.85–5.91; proportion main mode: 210.50, 22.01–2013.20) as well as with an increase in car share (HHI: 14.10, 4.42–44.96; number of modes: 2.57, 1.62–4.07; proportion main mode: 29.01, 5.16–163.30) (Table 7).

Exposure to the intervention predicted a decrease, but not an increase, in car mode share: individuals living 4 km from the busway were approximately twice as likely to reduce the share of their commuting journeys made entirely by car by more than 20% than those living 9 km away. There was a significant interaction between exposure to the intervention and baseline variability, in that individuals who had a greater level of variability at baseline were more likely to reduce their car share upon greater exposure to the intervention.

The sensitivity analyses revealed that with a less restrictive (0%) definition of change (i.e. defining *any* change in modal share as ‘change’), the effect of variability of baseline behaviour was stronger and the effect of exposure to the intervention was attenuated (Appendix C1). In the model with a more restrictive (30%) definition of change, variability was no longer significantly associated with a decrease in car use, although the interaction effect was strengthened. The findings in the other sensitivity tests were fairly similar to the results of the maximally adjusted model, but the associations did not always remain statistically significant. The effect of exposure to the intervention changed slightly in either direction after adding a measure of baseline variability to the model (Appendix C2).

## 4 Discussion and conclusion

### 4.1 Principle findings and their interpretation

We found that variability in the mode of transport used for commuting at baseline was associated with changes in modal split for commuting over time. All three indicators of variability in mode choice at baseline were significantly associated with an increase in the shares of commuting trips ‘made entirely by car’ and ‘involving any public transport’, and with a decrease in the shares of trips ‘made entirely by car’, ‘involving any public transport’ and ‘involving any active travel’. This indicates that on average individuals with higher levels of baseline variability were more likely to change their travel behaviour. This finding was largely robust to sensitivity analyses using a more restrictive threshold for change.

Although this study cannot reveal the exact cause, several potential explanations may underlie the associations observed. First, in agreement with existing conceptualisations, higher levels of variability may indicate that an individual is in an experimental phase and therefore open to changes (Jones and Sloman, 2003). Second, higher levels of variability indicate the use of a variety of modes, including active travel and public transport. This pattern of use may result in higher levels of self-efficacy (Bandura, 1986, Strecher et al., 1986) to use these modes of transport and thereby increase people’s responsiveness to a subsequent intervention. A third potential explanation is that some of the measured change over time may actually represent variability, possibly as a result of changes in accessibility needs. For example, individuals may have needed to commute to different locations at baseline and follow-up, resulting in different modal mixtures at the two time points. Further explorations (not presented) showed that at face value both modal shifts and random changes were present in the data. Our sensitivity tests with more conservative thresholds showed relative consistent results with the maximally adjusted models. This may indicate that the measured change is a combination of both change and random variability. Another possibility is that the results might partly reflect regression to the mean. People who show more variability at one time point are, all other things being equal, likely to show less variability at a second time point, and vice versa. However, although the outcome (change in mode share) and predictor (variability) were derived from the same survey question they are *different* measures, and the results are therefore unlikely to be entirely explicable by regression to the mean.

Individuals with higher levels of variation at baseline were more likely to increase their active travel share and reduce their car share with an increase in exposure to the intervention (shown by the significant interaction effects). These findings partially support our hypothesis that individuals who are more variable at baseline are more inclined to change if exposed to an intervention. The fact that the intervention produced a stronger change within this group may also indicate that individuals who show greater mode choice variability have a different decision-making style. We did not expect that variability or, in particular, the interaction between variability and exposure to predict a ‘negative’ response to the intervention. The association between baseline variability and an increase in car share and a decrease in the shares of trips involving active travel or public transport may be explained by a connection between variability and baseline use. Individuals with higher baseline variability may also be more likely to use all three modes of travel, and the use of a given mode at baseline increases the possibility of a change in modal split over time in either direction.

The association of exposure (proximity) to the intervention with changes in mode shares was hardly affected by the inclusion of baseline variability. This indicates that variability was not a confounder in the relationship between new infrastructure and travel behaviour, but ‘only’ an effect modifier and independent predictor of travel behaviour change.

We found only small differences between the different indicators of variability at the stage or trip level. In addition, other indicators we explored showed great similarity with the indicators we tested. This corroboration strengthens the inference that variability does indeed predict travel behaviour change, and suggests that the precise choice of variability measure to use in such analyses may not be critical.

### 4.2 Strengths and limitations

This study has provided evidence of an association of baseline variability with travel behaviour change. Key strengths of this study include the natural experimental study design, which allowed us to distinguish between variability at a given point in time and change over time. We also applied an individual measure of exposure to the intervention and controlled for multiple covariates to limit the possibility of alternative explanations. However, the study has several shortcomings. First, travel behaviour was self-reported and collected for only a seven-day period at each time point, which could bias the estimates of both variability and change. A longer duration of data collection would reduce the effects of occasional outliers in behaviour and would be more likely to pick up variability at baseline. However, imposing a greater burden on respondents in this way may increase the likelihood of dropout from a study. Second, variability can be measured in many ways and although we applied several measures together, as described above, all had shortcomings such as fixed boundaries and peaks in the data. Third, the study focussed solely on commuting, while the intervention could be expected to have had wider impacts on travel behaviour. However, the busway was expected to influence commuting journeys in particular, and the focus on commuting allowed us to examine a trip purpose with a relatively stable pattern in terms of location, travel time and frequency over the years; this seemed a particularly suitable focus for investigating the impact of variability on behaviour change. Fifth, we considered walking and cycling together in this study as active travel, and we also grouped bus and train use into public transport. Existing research has shown that the use of these modes is predicted by different factors. Separate analyses would have been interesting and could be pursued in future studies using larger datasets, but given the sample size and measured changes in mode choice in this study we decided to align the approach with that taken in previous papers and analyse these modes together. Fifth, the sample was relatively small and not entirely representative of the local population, and the findings cannot necessarily be generalised. Additional intervention studies that include an assessment of the amount of behaviour change predicted by variability would be necessary to confirm our findings.

### 4.3 Implications

The possibility that the measured change may partly reflect variability is important for other (intervention) studies of travel behaviour change. When defining and measuring behaviour change outcomes, researchers should keep in mind that these may be capturing variability instead, and consider conducting sensitivity tests using different definitions of ‘change’.

The significant interaction effect between variability and exposure to the intervention in predicting changes in active travel and car mode shares for commuting suggests that individuals with higher levels of variability may be more receptive to the intervention. The implication that interventions to promote active travel may be more effective in populations with higher levels of baseline variability could have important implications for practice. Further research on the predictors of baseline variability could help in understanding which groups would be more inclined to change their travel behaviour when presented with an intervention, and these groups could then be specifically targeted to increase the effectiveness of a given intervention. It would also be possible to investigate how variability can be increased, assuming that self-efficacy is the reason why higher levels of variability predict change. This knowledge could be used to design a two-step intervention, in which the first step would aim to increase variability as a prelude to a second, infrastructural intervention such as the busway evaluated in this study. This might be a more effective strategy for changing travel behaviour to improve population health.

## Authors’ contributions

DO led the design of the overall study in collaboration with others, see Acknowledgements. EH and DO conceived of the analysis for this paper and wrote the manuscript. EH conducted the analysis and drafted the manuscript. All authors read and approved the final manuscript. Other contributions are detailed in the Acknowledgements section.

## Figures and Tables

**Table 1:** Distribution of outcome variables (changes in commute mode share).

	Decrease of more than 20%		No change		Increase of more than 20%	
	*n*	%	*n*	%	*n*	%
Any active travel	48	10.6	332	73.6	71	15.7
Any public transport	39	8.6	373	82.7	39	8.6
Car	68	15.1	328	72.7	55	12.2

**Table 2:** Indicators of variability in mode choice.

**Indicator**	**Level**	**Applied transformation**	**Range**	**Mean (st.d.) or proportion**
HHI	Stage	1- value	0 to 0.9	0.26 (0.29)
Entropy	Stage		0–2	0.48 (0.55)
Number of modes (or combinations of modes) used	Trip		1–4	1.41 (0.64)
Using more than one mode	Trip		Yes/no	Yes=37.3%
No=62.7%
Share of the most commonly used mode (or combination of modes)	Trip	1-value	0 to 0.7	0.12 (0.17)

**Table 3:** Correlations of included variables.

	HHI	Number of modes (or combinations of modes) used	Share of the most commonly used mode (or combination of modes)
HHI	*1*		
Number of modes (or combinations of modes) used	*0.65*	*1*	
Share of the most commonly used mode (or combination of modes)	*0.64*	*0.80*	*1*

**Table 4:** Summary of participant characteristics and exposure to intervention.

		All participants at wave 1				Analysis sample (valid wave 1 and wave 4)				Participants in wave 1, but not wave 4 (drop out)^a^			
	
		*n*	%	Mean	St.d.	*n*	%	Mean	St.d.	*n*	%	Mean	St.d.
Distance from home to busway (km)		1155		6.6	7.8	466		6.5	7.9	659		6.7	7.7
Proximity to busway (-√km)		1155		−2.2	1.4	466		−2.1	1.4	659		−2.2	1.4
Commute distance (km)		1158		11.1	9.4	469		10.9	9.4	659		11.4	9.5
Moved home	No					359	76.4						
	Yes					111	23.6						
Moved workplace	No					357	76						
	Yes					113	24						
Gender	Male	367	31.5			157	33.4			205	30.9		
	Female	797	68.5			313	66.6			459	69.1		
Age	≤30	197	17			58	12.4			135	20.4		
	31–40	327	28.2			111	23.7			207	31.3		
	41–50	305	26.3			139	29.6			160	24.2		
	51–60	246	21.2			122	26			114	17.2		
	61+	85	7.3			39	8.3			46	6.95		
Education level	Degree	837	72.3			350	74.6			471	71.4		
	Less than degree	321	27.7			119	25.4			189	28.6		
Housing tenure	Not owner	319	27.5			103	22			204	30.9		
	Owner	840	72.5			365	78			457	69.1		
Driving licence	No	113	9.7			37	7.9			72	10.9		
	Yes	1049	90.3			432	92.1			591	89.1		
Access to a bicycle	No	182	15.7			63	13.5			191	28.8		
	Yes	974	84.3			404	86.51			473	71.2		
Children in household	No	820	70.5			324	68.9			473	71.2		
	Yes	344	29.6			146	31.1			191	28.8		
Physical health (PCS-8)		1156		53.7	6.3	468		53.9	6.3	658		53.6	6.4
Mental health (MCS-8)		1156		50.6	8.1	468		51.7	7.1	658		49.9	8.6
Difficulty walking	No	1143	98.5			464	98.9			651	98.3		
	Yes	18	1.6			5	1.1			11	1.7		
Type of settlement	Urban (>10,000)	767	66			316	67.4			427	64.3		
	Town & Fringe	226	19.4			80	17.1			144	21.7		
	Village, Hamlet & Isolated Dwellings	170	14.6			73	15.6			93	14.0		
Car parking at work	No	371	32.3			151	32.4			208	31.8		
	Yes, paid	351	30.6			143	30.7			201	30.7		
	Yes, free	427	37.2			172	36.9			245	37.5		
Car ownership	No car	175	15.0			57	12.1			112	16.9		
	One car	525	45.1			225	47.9			287	43.2		
	Two or more cars	464	39.9			188	40.0			265	39.9		

a The analysis sample was significantly different from the participants of wave 1 not included in analysis (due to dropout or other exclusion criteria) in terms of age, housing tenure, MCS-8, presence of a limiting health condition, and proportion of commuting trips made only by car at baseline.

**Table 5:** Association between baseline variability in mode choice and a change in active travel mode share for commuting.

		Measure of baseline variability					
		HHI		Number of modes		Proportion main mode	
		RRR	95% CI	RRR	95% CI	RRR	95% CI
Decrease in active travel share	Baseline variability	40.82***	**[10.63, 156.70]**	3.23***	**[2.04, 5.12]**	46.32***	**[8.10, 264.90]**
Exposure^a^	1.61*	**[1.06, 2.44]**	1.18	[0.87, 1.59]	1.20	[0.89, 1.62]
Interaction^b^	0.48	[0.17, 1.33]	–	–	1.06	[0.34, 3.23]

Increase in active travel share	Baseline variability	5.48**	**[1.71, 17.52]**	2.95***	**[1.81, 4.79]**	106.40***	**[14.97, 756.40]**
Exposure^a^	1.62**	**[1.16, 2.26]**	1.75***	**[1.27, 2.42]**	1.68**	**[1.21, 2.35]**
Interaction^b^	3.34**	**[1.41, 7.89]**	–	–	6.97**	**[1.63, 29.88]**

Multinomial logistic regression with ‘no change’ as the reference outcome category.

The models are adjusted for: gender, age, education level, housing tenure, access to a bicycle, driving licence, car ownership, commute distance, physical health (PCS-8), mental health (MCS-8), limiting health condition, type of settlement and car parking at work.

Values tabulated are relative risk ratios (RRR) and 95% confidence intervals (95% CI).

a Exposure to the intervention. Exposure to the busway was defined as the negative square root of the distance from home to busway.

b Interaction between exposure to the intervention and the measure of baseline variability of mode choice.

* *p*<0.05.

** *p*<0.01.

*** *p*<0.001.

**Table 6:** Association between baseline variability in mode choice and a change in public transport mode share for commuting.

		Measure of baseline variability					
		HHI		Number of modes		Proportion main mode	
		RRR	95% CI	RRR	95% CI	RRR	95% CI
Decrease in public transport share	Baseline variability	82.83^***^	**[15.17, 452.40]**	3.64^***^	**[2.17, 6.11]**	50.50^***^	**[7.14, 357.30]**
Exposure^a^	1.06	[0.73, 1.54]	0.94	[0.66, 1.36]	0.94	[0.66, 1.35]
Interaction^b^						

Increase in public transport share	Baseline variability	1.71	[0.49, 6.03]	1.78^*^	**[1.05, 3.04]**	8.29^*^	**[1.18, 58.57]**
Exposure^a^	1.54^*^	**[1.11, 2.15]**	1.51^*^	**[1.09, 2.10]**	1.52^*^	**[1.09, 2.11]**
Interaction^b^						

Multinomial logistic regression with ‘no change’ as the reference outcome category.

The models are adjusted for: gender, age, education level, housing tenure, access to a bicycle, driving licence, car ownership, commute distance, physical health (PCS-8), limiting health condition and having a child in the household.

Values tabulated are relative risk ratios (RRR) and 95% confidence intervals (95%CI).

^a^ Exposure to the intervention. Exposure to the busway was defined as the negative square root of the distance from home to busway.

^b^ Interaction between exposure to the intervention and the measure of baseline variability of mode choice.

^*^p<0.05.

^**^p<0.01.

^***^p<0.001.

**Table 7:** Association between change in car mode share for commuting and baseline variability in mode choice.

		Measure of baseline variability					
		HHI	HHI	Number of modes		Proportion main mode	
		RRR	RRR	RRR	RRR	RRR	95%CI
Decrease in car share	Baseline variability	6.00*	**[1.51, 23.79]**	3.31***	**[1.85, 5.91]**	210.50***	**[22.01, 2013.20]**
Exposure^a^	1.85**	**[1.26, 2.72]**	2.12***	**[1.44, 3.11]**	1.85**	**[1.27, 2.70]**
Interaction^b^	7.50***	**[2.52, 22.34]**	1.60*	**[1.04, 2.44]**	18.23**	**[3.02, 110.20]**

Increase in car share	Baseline variability	14.10***	**[4.42, 44.96]**	2.57***	**[1.62, 4.07]**	29.01***	**[5.16, 163.30]**
Exposure^a^	1.22	[0.86, 1.73]	1.13	[0.83, 1.55]	1.10	[0.81, 1.49]
Interaction^b^	0.93	[0.41, 2.10]	1.17	[0.88, 1.56]	2.22	[0.71, 6.92]

Multinomial logistic regression with ‘no change’ as the reference outcome category.

The models are adjusted for: gender, age, education level, access to a bicycle, driving licence, car ownership, commute distance, physical health (PCS-8), mental health (MCS-8), limiting health condition, type of settlement, having change work address and car parking at work.

Values tabulated are relative risk ratios (RRR) and 95% confidence intervals (95%CI).

a Exposure to the intervention. Exposure to the busway was defined as the negative square root of the distance from home to busway.

b Interaction between exposure to the intervention and the measure of baseline variability of mode choice.

* *p*<0.05.

** *p*<0.01.

*** *p*<0.001.

## Footnotes

1 The distances chosen serve as an example. Any other distance combination corresponding with the square root transportation could serve as an example, e.g. 1 km instead of 4 km.

## References

[bib1] Aarts H., Verplanken B., Van Knippenberg A. (1998). Predicting behavior from actions in the past: repeated decision making or a matter of habit?. J. Appl. Soc. Psychol..

[bib2] Atkins, 2004. Transport Assessment. Cambridgeshire Guided Busway. Cambridgeshire Country Council, Cambridge.

[bib3] Bamberg S., Schmidt P. (2003). Incentives, morality, or habit? Predicting students’ car use for university routes with the models of Ajzen, Schwartz, and Triandis. Environ. Behav..

[bib4] Bandura A. (1986). The explanatory and predictive scope of self-efficacy theory. J. Soc. Clin. Psychol..

[bib5] Bibby P., Shephard J. (2004). Developing a New Classification of Urban and Rural Areas for Policy Purposes – The Methodology.

[bib6] Chief Medical Officers, 2011. Start Active, Stay Active: A Report on Physical Activity from the Four Home Countries’. Chief Medical Officers. Department of Health, London.

[bib7] De Bruijn G.J., Gardner B. (2011). Active commuting and habit strength: an interactive and discriminant analyses approach. Am. J. Health Promot..

[bib8] Ewing R., Cervero R. (2010). Travel and the built Environment, a meta analyses. J. Am. Plann. Assoc..

[bib10] Gärling T., Axhausen K.W. (2003). Introduction: habitual travel choice. Transportation.

[bib11] Goodman A., Sahlqvist S., Ogilvie D. (2013). Who uses new walking and cycling infrastructure and how? Longitudinal results from the UK iConnect study. Prev. Med..

[bib12] Goodman A., Sahlqvist S., Ogilvie D. (2014). New walking and cycling routes and increased physical activity: one- and 2-year findings from the UK iConnect study. Am. J. Public Health.

[bib13] Handy S.L., Boarnet M.G., Ewing R., Killingsworth R.E. (2002). How the built environment affects physical activity views from urban planning. Am. J. Prev. Med..

[bib14] Heinen E., Panter J., Mackett R., Ogilvie D. (2015). The impact of new transport infrastructure on active and car commuting: a natural experiment study. Int. J. Behav. Nutr. Phys. Act..

[bib15] Heinen E., Panter J., Dalton A., Jones A., Ogilvie D. (2015). Sociospatial patterning of the use of new transport infrastructure: walking, cycling and bus travel on the Cambridge Guided Busway. J. Transp. Health. J. Transp. Health.

[bib16] Heinen E., Chatterjee K. (2015). The same mode again? An exploration of mode choice variability in Great Britain using the National Travel Survey. Accept. Publ. Transp. Res. Part A.

[bib17] Hooper P., Giles-Corti B., Knuiman M. (2014). Evaluating implementation and active living impacts of a state government planning designed to create walkable neighborhoods in Perth, Western Australia. Am. J. Health Promot..

[bib18] Hymowitz N., Cummings K.M., Hyland A., Lynn W.R., Pechacek T.F., Hartwell T.D. (1997). Predictors of smoking cessation in a cohort of adult smokers followed for five years. Tob. Control.

[bib19] Jones, P.M., Sloman, L., 2003. Encouraging behavioural change through marketing and management: what can be achieved? Paper Presented at the 10th International Conference on Travel Behaviour Research, Lucerne, Switzerland.

[bib20] McCormack G.R., Shiell A. (2011). In search of causality: a systematic review of the relationship between the built environment and physical activity among adults. Int. J. Behav. Nutr. Phys. Act..

[bib21] National Institute for Health and Care Excellence (Nice), 2014. Physical activity and the environment Evidence Update April 2014 Evidence Update 57. 2014. National Institute for Health and Care Excellence, London.

[bib22] Ogilvie D., Griffin S., Jones A., Mackett R., Guell C., Panter J. (2010). Commuting and health in Cambridge: a study of a ‘natural experiment’ in the provision of new transport infrastructure. BMC Public Health.

[bib23] Office for National Statistics (ONS). Census 2011. 2011, via 〈http://www.ons.gov.uk/ons/guide-method/census/2011/index.html〉. (accessed 31.03.14).

[bib24] Paavola M., Vartiainen E., Haukkala A. (2004). Smoking, alcohol use, and physical activity: a 13-year longitudinal study ranging from adolescence into adulthood. J. Adolesc. Health.

[bib25] Panter, J., Heinen, E., Mackett, R., Ogilvie, D, Impact of new transport infrastructure on walking, cycling and physical activity. Am. J. Prev. Med. 2016, in press. 10.1016/j.amepre.2015.09.021 PMC4712020 26585051

[bib26] Panter J., Griffin S., Jones A., Mackett R., Ogilvie D. (2011). Correlates of time spent walking and cycling to and from work: baseline results from the commuting and health in Cambridge study. Int. J. Behav. Nutr. Phys. Act..

[bib27] Prochaska J.O., DiClemente C.C., Norcross J.C. (1992). In search of how people change. Applications to addictive behaviors. Am. Psychol..

[bib28] Prochaska J.O., Velicer W.F. (1997). The transtheoretical model of health behavior change. Am. J. Health Promot..

[bib29] Rhoades S.A. (1993). The Herfindahl-Hirschman Index. Fed. Reserve Bull..

[bib30] Strecher V.J., McEvoy DeVellis B., Becker M.H., Rosenstoc I.M. (1986). The role of self-efficacy in achieving health behavior change. Health Educ. Behav..

[bib31] Susilo Y.O., Axhausen K.W. (2014). Repetitions in individual daily activity-travel-location patterns: a study using the Herfindahl–Hirschman Index. Transportation.

[bib32] Sutton, A., 2002. Health Behaviour: Psychological Theories. 〈http://userpage.fu-berlin.de/~schuez/folien/Sutton.pdf〉

[bib33] Ware J., Kosinski M., Dewey J., Gandek B. (2008). How to Score and Interpret Single-item Health Status Measures: A Manual for Users of the SF-8 ™ Health survey.

[bib34] Woodcock J., Edwards P., Tonne C., Armstrong B.G., Ashiru O., Banister D. (2009). Public health benefits of strategies to reduce greenhouse-gas emissions: urban land transport. Lancet.

